# Unfolding the Interactions between Endoplasmic Reticulum Stress and Oxidative Stress

**DOI:** 10.3390/antiox12050981

**Published:** 2023-04-22

**Authors:** Gideon Ong, Susan E. Logue

**Affiliations:** 1 Department of Human Anatomy and Cell Science, Rady Faculty of Health Sciences, University of Manitoba, Winnipeg, MB R3E 0J9, Canada; 2 CancerCare Manitoba Research Institute, Winnipeg, MB R3E 0V9, Canada; 3 The Children’s Hospital Research Institute of Manitoba (CHRIM), Winnipeg, MB R3E 3P4, Canada

**Keywords:** antioxidants, endoplasmic reticulum, reactive oxygen species, unfolded protein response

## Abstract

Oxidative stress is caused by an imbalance in cellular redox state due to the accumulation of reactive oxygen species (ROS). While homeostatic levels of ROS are important for cell physiology and signaling, excess ROS can induce a variety of negative effects ranging from damage to biological macromolecules to cell death. Additionally, oxidative stress can disrupt the function of redox-sensitive organelles including the mitochondria and endoplasmic reticulum (ER). In the case of the ER, the accumulation of misfolded proteins can arise due to oxidative stress, leading to the onset of ER stress. To combat ER stress, cells initiate a highly conserved stress response called the unfolded protein response (UPR). While UPR signaling, within the context of resolving ER stress, is well characterised, how UPR mediators respond to and influence oxidative stress is less defined. In this review, we evaluate the interplay between oxidative stress, ER stress and UPR signaling networks. Specifically, we assess how UPR signaling mediators can influence antioxidant responses.

## 1. Introduction

Reactive oxygen species (ROS) are a set of highly unstable oxygen-containing molecules or free radicals. The main forms of ROS generated under physiologic conditions include hydrogen peroxide (H_2_O_2_), superoxide anion (O_2_^•−^) and hydroxyl radicals (^•^OH). In cellular physiology, low levels of ROS are vital for cell signaling, but high levels constitute a threat by potentially damaging DNA, proteins and lipids. Therefore, cells must have strategies to carefully control ROS levels. Counterbalancing ROS is achieved via antioxidants; these compounds or redox systems (including glutathione and thioredoxin systems) convert ROS into stable molecules, thereby blocking their damaging effects. The maintenance of optimal cellular ROS involves a careful balance of ROS production versus antioxidant-controlled neutralisation. The disruption of this balance triggers ROS accumulation, a state known as oxidative stress, which if not resolved jeopardizes cellular health.

The mitochondria are the most recognised cellular source of ROS. During oxidative phosphorylation, a small percentage of electrons leak from complex I, II and III of the electron transport chain to form superoxide anions [1]. These ROS, while potentially harmful, also act as important signaling molecules. Mitochondrial ROS have been reported to induce PI3K signaling, activate hypoxia inducible factors (HIFs) and modify metabolism [2].

While the mitochondria are the most recognised cellular source of ROS, the endoplasmic reticulum (ER) is also a significant contributor, where it is estimated to account for around 25% of total ROS generation [3]. Similar to the mitochondria, within the ER, ROS generation is a by-product of an important biochemical process, namely protein folding. Given the large volume of ROS produced within the ER, effective countermeasures are essential to maintain redox balance. In this review article, we focus upon the ER, outlining the relationship between ER function, oxidative stress and the unfolded protein response (UPR), an ER-specific stress response pathway.

## 2. The Endoplasmic Reticulum and ROS Production

The ER is comprised of a series of tubules and sheet-like structures spanning throughout the cytoplasm and serves critical cellular functions including lipid and steroid production, calcium storage and protein synthesis, folding and transport [4,5]. Disulfide bond formation is one of the most common post-translational modifications occurring within the ER and is essential for the generation of stable, correctly folded proteins. To facilitate protein folding, the ER requires high levels of calcium (to support ER chaperone protein function) and must maintain a highly oxidizing environment to enable disulfide bond formation [6,7]. 

The protein disulfide isomerase (PDI) family of proteins play a central role in forming disulfide bridges. These calcium-binding chaperones possess oxidoreductase and isomerase activity, mediated by four thioredoxin-like domains a, b, a’, b’, with a and a’ containing a Cys-X-X-Cys sequence enabling disulfide oxidoreductase activity. While the b and b’ domains lack catalytic activity, they contribute to the binding of protein substrates [8]. Oxidized PDI is the working unit responsible for extracting electrons from the cysteine residues in newly synthesized proteins, thus creating an intramolecular disulfide bridge, a process that results in PDI reduction [9]. For further rounds of disulfide bond formation, reduced PDI requires re-oxidization, which is facilitated by several redox pathways. Endoplasmic reticulum oxidoreductase 1 (ERO1) replenishes oxidized PDI, but in doing so generates ROS by using O_2_ as an electron acceptor, which forms the reactive oxygen species H_2_O_2_ [9,10]. Alternatively, oxidized peroxiredoxin 4 (PRDX4), and glutathione peroxidase 7 and 8 (GPx7 and GPx8) can accept electrons to regenerate re-oxidized PDIs for subsequent rounds of protein disulfide bond formation. PRDX4, GPx7 and GPx8 also act as important ER-resident antioxidants as they utilize ERO1-generated H_2_O_2_ as an electron acceptor during cyclic redox reactions to form H_2_O [11,12,13]. Collectively, these enzymes are critical for ensuring the complete reduction of O_2_ to H_2_O and the maintenance of redox balance within the ER during the process of oxidative protein folding.

Protein disulfide bond formation can also be catalyzed through enzymatic reactions that do not depend on ERO1. This includes the vitamin K epoxide reductase (VKOR) pathway. VKOR can undergo a cyclic redox reaction by accepting electrons from thioredoxin-like proteins (which catalyze protein disulfide bond formation) and by reducing vitamin K epoxides [14]. The VKOR pathway has been described to be important in managing ER redox levels during oxidative protein folding [15]. Quiescin sulfhydryl oxidase (QSOX) is another enzyme that can directly catalyze protein disulfide bond formation through the reduction of O_2_ into H_2_O_2_ [16]. However, these pathways are not as well characterised and further investigation is required to assess their overall impact on ER ROS.

ER-derived ROS can also originate from enzymatic reactions beyond protein folding. The cytochrome P450 (CYP) family of enzymes are known for their role in lipid and drug metabolism, and many are localized to the endoplasmic reticulum of hepatocytes [17]. CYP catalyzes the hydroxylation of a hydrocarbon substrate with the assistance of electron donors in the presence of oxygen. However, ROS may leak during the intermediary steps of the reaction (O_2_^•−^ and H_2_O_2_), thus contributing to endoplasmic reticulum ROS levels [18]. CYP2A1 and CYP2E1 have been characterised as ‘leaky’ enzymes which contribute to ROS production [19,20]. Meanwhile, the stimulation of hepatocytes with free fatty acids elevated ROS levels through CYP4A11 [21]. CYP2C9 was also demonstrated to generate H_2_O_2_, which was reduced upon interaction with cytochrome b_5_ [22]. 

While the ER has defense strategies, if ROS levels exceed the buffering capacity of ROS scavengers, ER function becomes impeded. Increased ROS can result in excess oxidation or hyperoxidation of ER-resident proteins, altering their conformation and function. For example, PRDX4 can be irreversibly inactivated through overoxidization into its sulfonic acid form (-SO_3_H) under high H_2_O_2_ levels [23,24]. Furthermore, perturbances within ER redox homeostasis disrupt the conditions required to support disulfide bond formation, thus negatively affecting protein folding [25]. This can result in the buildup of misfolded or unfolded proteins within the ER lumen, a state commonly referred to as ER stress. To combat ER stress, cells trigger the activation of the UPR. The UPR aims to restore ER homeostasis by promoting the folding of proteins that can be refolded, while initiating the degradation of those beyond repair via a process referred to as ER-associated degradation (ERAD). The UPR and its constituent signaling pathways have been well characterised over the past few decades.

## 3. The Unfolded Protein Response

**Figure 1:**
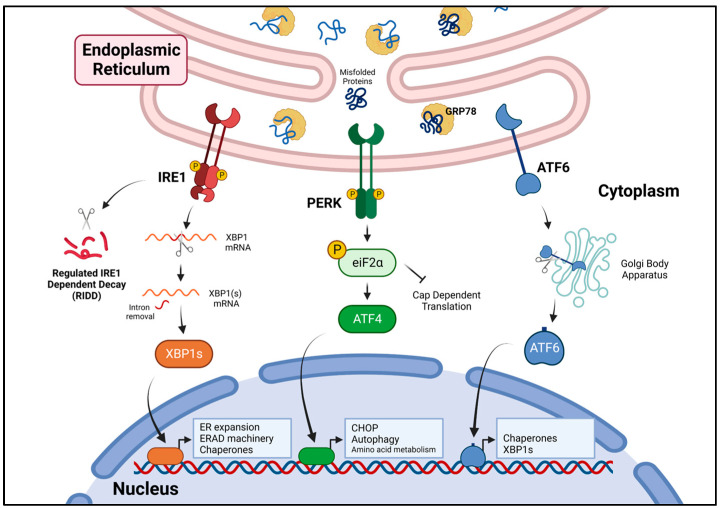
The unfolded protein response and its role in ER stress. As misfolded proteins accumulate within the endoplasmic reticulum lumen, GRP78 dissociates from IRE1, PERK and ATF6 allowing for their activation. IRE1 RNase cleaves its downstream target XBP1 mRNA which becomes religated to form XBP1s mRNA encoding for the transcription factor XBP1s. PERK phosphorylates its downstream target eiF2α which suppresses CAP-dependent translation while allowing for selective translation of ATF4. Upon ER stress, ATF6 translocates to the Golgi where it becomes cleaved into its active form. The initiation of the UPR signaling pathways enhances expression of genes that enable adaptation and resolution of ER stress. Created with BioRender.com.

The UPR is a collective term for a series of complex, dynamic signaling processes controlled by three receptors, inositol requiring enzyme-1 (IRE1), protein kinase R-like endoplasmic reticulum kinase (PERK) and activating transcription factor 6 (ATF6). These receptors, anchored on the ER membrane, are single-pass transmembrane proteins with either cytosolic carboxyl termini (IRE1, PERK) or a cytosolic amino terminus (ATF6). Under non-stress conditions, IRE1, PERK and ATF6 remain in an “off” position via the binding of their luminal terminus to the ER chaperone glucose regulated protein 78 (GRP78) [26]. The accumulation of unfolded proteins within the ER lumen breaks the association with GRP78 (as GRP78 preferentially binds unfolded proteins), permitting IRE1, PERK and ATF6 to undergo changes facilitating their activation (Figure 1). 

### 3.1. IRE1 Signaling

IRE1 consists of two isoforms, IRE1α (hereafter referred to as IRE1) which is ubiquitously expressed, and IRE1β whose expression is restricted to epithelial cells lining mucosal surfaces such as the intestine [27,28]. IRE1 is a type I transmembrane ER-anchored receptor protein consisting of cytosolic Serine/Threonine kinase and the ribonuclease domain [29]. Following the dissociation of GRP78, IRE1 homodimerizes and transautophosphorylates, facilitating the activation of its RNase activity [30,31]. Through its RNase activity, IRE1 cleaves X-box binding protein 1 (XBP1) mRNA, facilitating the removal of a 26-nucleotide intron [32]. The RNA 2’,3’-cyclic phosphate and 5’-OH ligase (RTCB) re-ligates cleaved XBP1 mRNA generating spliced XBP1 (XBP1s), which, when translated, produces the transcription factor XBP1s [33]. XBP1s binds to ER stress response element (ERSE), ER stress response element II (ERSE II) and unfolded protein response element (UPRE) sequences [34,35]. This upregulates the expression of genes encoding proteins that (A) promote protein refolding (ER chaperone proteins), (B) aid in the destruction of those proteins beyond repair (components of the ER associated degradation (ERAD) machinery) and (C) encode proteins that facilitate the expansion of the ER, thus increasing protein folding capacity [36,37,38]. In addition to splicing XBP1, IRE1 RNase activity has the capacity to cleave other RNA targets in a process known as regulated IRE1 dependent decay (RIDD) [39,40]. Initial studies demonstrated that RIDD targets contained a consensus sequence (CTGCAG) and a secondary structure similar to that found in XBP1 mRNA [41]. However, recent studies suggest that RIDD may be a more complex process, with the oligomerization/phosphorylation status of IRE1 influencing the selection of RIDD targets. Under relatively modest activation, IRE1 selectively targets substrates exhibiting the preferred consensus sequence. However, as IRE1 activation increases, RIDD activity becomes more promiscuous resulting in widespread mRNA degradation, a process that has been coined RIDD Lacking Endomotif, or RIDDLE [42]. Initial studies linked RIDD to the destruction of mRNAs encoding ER bound proteins. This suggested that RIDD aids a stressed ER by preventing the production of proteins destined for the ER [39]. As our knowledge of RIDD has increased, so too has our understanding of its functional outcomes, both within the specific setting of ER stress and its broader physiological impact. IRE1-RIDD has been demonstrated to maintain cell viability during ER stress by degrading death receptor 5 (DR5), thereby neutralising CHOP-mediated increases in DR5 [43]. The attenuation of RIDD activity, as observed during excessive ER stress, halts DR5 degradation resulting in DR5 mediated cell death [44]. In terms of its wider biological implications, RIDD activity contributes to a wide range of physiological processes ranging from lipid metabolism and insulin production to innate and adaptive immunity [45,46,47].

While IRE1 RNase activity has been the focus of most research, the kinase activity of IRE1 can also trigger downstream signaling. Although not extensively characterised, IRE1 kinase activity has been demonstrated to recruit TNF receptor-associated factor 2 (TRAF2) leading to c-Jun N terminal Kinase (JNK) signaling [48]. As JNK activation can phosphorylate BCL-2 family members, IRE1-JNK signaling may promote a shift towards pro-death signaling by increasing the activity of pro-apoptotic BCL-2 family members while reducing that of anti-apoptotic BCL-2 family members [49].

### 3.2. PERK Signaling

PERK, similar to IRE1, is a type I transmembrane ER-anchored receptor protein which undergoes dimerization and transautophosphorylation following the dissociation of GRP78, resulting in the activation of its cytosolic kinase domain [50]. Active PERK phosphorylates Ser-51 of eukaryotic translation initiation factor 2A (eIF2α), preventing interaction of eIF2α with the guanidine exchange factor eukaryotic translation initiation factor 2B (eIF2B), thereby blocking the assembly of the translational pre-initiation complex and ultimately preventing cap-dependent translation [51,52]. This pause in translation reduces the protein load placed upon the ER and by doing so offers the cell an opportunity to deal with the backlog of unfolded/misfolded proteins. While this translational block is widespread, it is not absolute. Due to the presence of upstream open reading frames and internal ribosomal entry sites, specific genes, such as activating transcription factor 4 (ATF4), are selectively translated under these conditions [53]. Increased ATF4 expression elevates downstream target genes implicated in a wide range of processes including amino acid metabolism and autophagy [54,55]. The transcription factor CCAAT/enhancer binding proteins-homologous protein (CHOP) is also upregulated by ATF4 [56]. Increased CHOP expression has been linked to a shift towards pro-apoptotic outcomes, both by direct regulation of downstream pro-apoptotic target genes and indirectly via the re-establishment of cap-dependent translation through the induction of growth arrest and DNA damage-inducible protein 34 (GADD34) which de-phosphorylates eIF2α [57,58,59].

### 3.3. ATF6 Signaling

Activating transcription factor 6 is a member of the ATF/CREB family of transcription factors. In an unstressed ER, owing to the presence of intra- and inter-disulphide bonds between conserved cysteine residues within the luminal domain, ATF6 exists in monomeric, dimeric and oligomeric forms [60]. During ER stress, ATF6 translocates to the Golgi apparatus where Site-1 and Site-2 proteases cleave it, generating the active transcription factor ATF6N [61]. For many years, precisely how the dissociation of Grp78 instigates a shift in ATF6 localisation from the ER to the Golgi apparatus was unclear. Recent studies suggest that the redox state of ATF6 affects its ability to undergo translocation and processing. Oka and colleagues demonstrated that during the induction of ER stress, ATF6 dimers linked by a C467-467 disulfide bond are preferentially translocated to the Golgi apparatus. Overexpression of the ER resident oxidoreductase ERp18 diminished the formation of ATF6 dimers (via reduction) and processing of ATF6 to ATF6N, whereas the loss of ERp18 improved the kinetics of ATF6 dimerization [62]. This observation suggests that the mechanisms governing ATF6 translocation and cleavage within the Golgi apparatus are complex, inferring a role of ERp18 in regulating this process. Upon cleavage, the N-terminal portion of ATF6 (ATF6N) is released and translocates to the nucleus where it binds to cis-regulatory elements, including ERSE I, ERSE II and UPRE promoter sequences [34,63,64]. ATF6 upregulates ER-associated genes including *XBP1*, thereby supporting IRE1 signaling and ER quality control proteins [37,65].

### 3.4. The Intersection of Oxidative Stress, ER Stress, and the Unfolded Protein Response

Given that protein folding is influenced by the redox state of the ER, it should be no surprise that ER stress and oxidative stress are two closely associated events. Indeed, UPR activation and increased ROS levels can coincide during the onset of stress. This phenomenon can be observed by certain thiol-containing compounds such as dithiothreitol (DTT), which disrupts the process of oxidative protein folding. Accordingly, DTT treatment initiates the UPR; however, increased ROS levels were also observed [66]. Similarly, α-lipoic acid has been reported to induce ROS production and UPR activation in hepatoma cells [67]. Tunicamycin and thapsigargin, which are classical well-known inducers of ER stress/UPR initiation, were also demonstrated to increase ROS levels [68,69]. These findings suggest that a relationship exists between oxidative stress and ER stress. However, the mechanisms that link these two stressors are rather complex.

**Figure 2:**
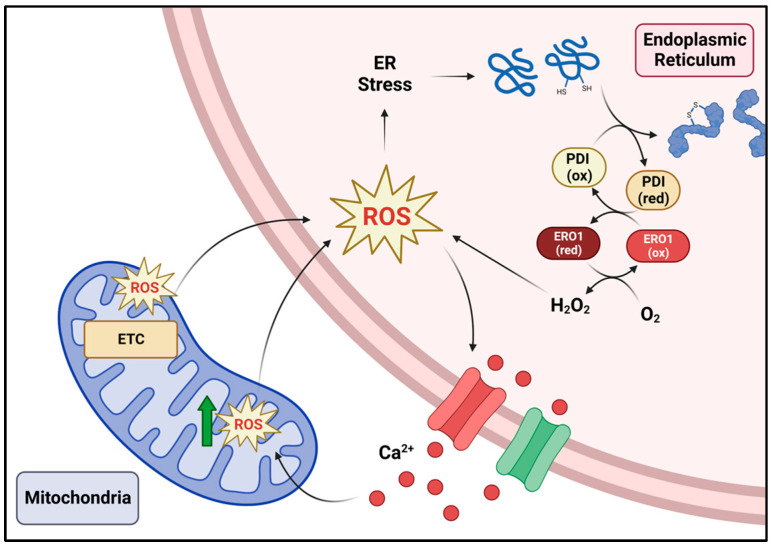
Both the endoplasmic reticulum and mitochondria are major sources of cellular ROS. If ROS levels increase, Ca^2+^ release by the ER occurs and can stimulate further ROS production in the mitochondria. This in turn contributes to further ER stress and oxidative stress in the ER. A vicious feedback loop occurs where ER stress aggravates oxidative stress and vice versa. Created with BioRender.com.

Increasing the levels of ROS can exacerbate ER stress and trigger downstream signaling events within the ER. ROS-sensitive Ca^2+^ channels inositol-1,4,5-trisphosphate receptor (IP3R) and ryanodine receptors (RyR) on the ER membrane can be activated during oxidative stress to trigger the release of Ca^2+^ stores [70]. Regions of the ER that interact with the mitochondria, known as mitochondria-associated ER membranes or MAMs, facilitate the uptake of ER-Ca^2+^ within the mitochondria, resulting in increased metabolism which stimulates further ROS production [71]. Increased mitochondrial ROS in turn can leak to the ER, further aggravating ER stress. Additionally, components of the UPR also directly contribute to oxidative stress. For example, the transcription factor CHOP can upregulate ERO1 which further potentiates the reduction of oxygen into hydrogen peroxide during oxidative folding [72]. Subsequently, increased ERO1 expression and accumulation of H_2_O_2_ enhance the activation of IP3R, triggering additional Ca^2+^ movement from the ER to the mitochondria thereby further exacerbating oxidative stress [73]. High H_2_O_2_ levels can also result in superoxidation of PRDX4, thus resulting in its inactivation [24]. Ultimately, these events support a vicious cycle where increased ROS triggers the release of Ca^2+^; this in turn stimulates mitochondrial ROS production which feeds back into the ER triggering Ca^2+^ release and further aggravating ER stress (Figure 2). If not controlled, the concurrent stressors will result in cell death. Perhaps not surprisingly, UPR mediators have been linked to signaling pathways aimed at alleviating oxidative stress and ultimately the restoration of ER homeostasis. 

## 4. Regulation of Oxidative Stress by the Unfolded Protein Response

### 4.1. PERK in Response to Oxidative Stress

In 2003, a critical link between PERK and the master regulator of the oxidative stress response, nuclear factor-erythroid 2 related factor 2 (NRF2), was identified [74]. NRF2 is a Cap N’ Collar (CNC) and b-Zip transcription factor which binds to and promotes the activation of genes containing an antioxidant response element, including components of the thioredoxin and glutathione systems [75,76]. Under homeostatic conditions, the Kelch-like ECH-associated protein 1 (KEAP1) binds to the NRF2-ECH homology (Neh2) domain of NRF2. This interaction brings NRF2 into the KEAP1–Cul3–E3 ubiquitin ligase complex where it is ubiquitinated, leading to its degradation via the 26S proteasome [77]. During oxidative stress, NRF2 binding to KEAP1 is disrupted resulting in NRF2 stabilisation and the upregulation of NRF2 target genes, thereby boosting antioxidant responses. The disruption of KEAP1–NRF2 binding during oxidative stress is achieved via several mechanisms. Cysteine residues (Cys226/613/622/624) present on KEAP1 act as sensors for H_2_O_2_ becoming oxidized by H_2_O_2_ to form intramolecular disulfide bonds, thus preventing NRF2 binding [78]. Additionally, PERK-dependent signaling can also contribute to NRF2 stabilisation. PERK-mediated phosphorylation of NRF2 on Thr-80 prevents NRF2:KEAP1 complex formation, thereby stabilising NRF2 and, through this, enabling cellular adaption to oxidative stress [74,79]. The PERK–NRF2 axis can interact with downstream targets including hypoxia-inducible factor 1α (HIF1α), and can upregulate the antioxidants hemeoxygenase 1 (HO-1) and NAD(P)H:quinone oxidoreductase 1 (NQO1) [80,81].

PERK can also indirectly support NRF2 activation via upregulation of ATF4. Sarcinelli et al. demonstrated that ATF4 upregulates the *Nrf2* transcript by binding to the C/ebp-Atf response element (CARE) *cis* regulatory element within the *Nrf2* promoter [82]. This contributed to an increase in antioxidant response genes including heme-oxygenase 1 (*HO1*), an enzyme involved in producing the antioxidant bilirubin, and glutamate-cysteine ligase-catalytic subunit (*GCLC*), the subunit of the rate-limiting enzyme for glutathione (antioxidant) synthesis. ATF4 can also bind directly with NRF2 to form a heterodimer and upregulate antioxidant genes containing the *cis* regulatory antioxidant response element, stress response element, and amino acid response element to aid in suppressing oxidative stress [83,84]. 

PERK signaling through the PERK–p-eIF2α–ATF4 axis also supports oxidative stress resistance by upregulating genes involved in acid transport and glutathione biosynthesis. Harding et al. demonstrated that mouse embryonic fibroblasts (MEF) lacking PERK or ATF4 expression had increased sensitivity to oxidative stress and reduced expression of *Glyt1* (glycine transporter 1) and *SLC3A2* (heavy chain of x^−^_c_ cystine/glutamate exchanger) [54]. These genes express transporters responsible for the import of glycine and cysteine, which are important precursors for glutathione. PERK–p-eIF2α–ATF4 was also shown to upregulate *GCLC*, *CTH* (cystathionine gamma-lyase, an enzyme involved in cysteine synthesis) and *SLC7A11* (solute carrier family 7 member 11, light chain of x^−^_c_ cysteine/glutamate antiporter) in MDA-MB-231 cells which resulted in increased amounts of reduced glutathione and decreased ROS levels [85]. Thus, PERK–p-eIF2α–ATF4-mediated increases in amino acid transport and metabolism are important for mitigating oxidative stress. 

Conversely, the ATF4 target CHOP has been reported to contribute to increased oxidative stress by upregulating ERO1, thus leading to a heightened production of H_2_O_2_ and increased apoptosis [72]. Meanwhile, the deletion of *CHOP* lowered ERO1 expression and oxidative stress in diabetic mouse models [86]. 

While PERK’s function is mostly associated with UPR signaling via its kinase activity, it also has important structural roles, acting as a tethering protein within MAMs which also has implications in regulating oxidative stress. PERK-deficient MEFs exhibit decreased MAM formation, thereby reducing Ca^2+^ and ROS signaling from the ER to the mitochondria, ultimately suppressing ROS-induced apoptosis [87]. Similarly, in rat models of diabetic cardiomyopathy, PERK knockdown decreased ROS-induced apoptosis [88]. More recently, Bassot et al. have examined the PERK interactome and identified ERO1 as a PERK binding partner [89]. During the early stages of ER stress, PERK and ERO1 rapidly complex at mitochondrial ER contact sites, an interaction that supports Ca^2+^ flux between the two organelles, thus helping to maintain bioenergetics and suppress oxidative stress. These findings underscore the importance of PERK enrichment at MAMs to facilitate effective communication between the ER and the mitochondria for the regulation of oxidative stress. 

### 4.2. IRE1 in Response to Oxidative Stress

IRE1-mediated signaling has been implicated in oxidative stress responses in various ways, both dependent and independent of its RNase activity. Guerra-Moreno et al. demonstrated a non-canonical activation of IRE1 in yeast (*Saccharomyces cerevisiae*) by sulfenylation of conserved cysteine residues present within the activation loop (C832 for yeast), activated antioxidant responses and reduced oxidative stress [90]. Similarly, Hourihan et al. described a unique mechanism where increased ROS production through the ER or mitochondria results in downstream activation of SKN-1 (*C. elegans*) and NRF2 (human) through IRE1. They demonstrated that the increased presence of ER ROS drives the sulfenylation of cysteine residues present within the activation loop of IRE1 [91]. In *C. elegans*, sulfenylated IRE1 recruits TRF1/NSY-1 (TRAF2/ASK1 in humans) which is required for subsequent phosphorylation of p38 MAPK signaling and downstream activation of NRF2 in a KEAP1 independent manner. Similarly, in human (HepG2) cells, IRE1 sulfenylation induced p38/NRF2 activation, highlighting the conservation of IRE1 antioxidant responses across species. IRE1 sulfenylation was mutually exclusive of IRE1 phosphorylation, as the induction of oxidative stress blocked XBP1s expression, while triggering ER stress repressed P-p38 expression [91]. 

Classical downstream signaling through IRE1-XBP1s can also influence oxidative stress. Fink et al. demonstrated that XBP1s upregulates Krüppel-like factor 9 (KLF9) during high levels of ER stress. Increased KLF9 amplifies both ER stress and oxidative stress through increased ER Ca^2+^ release, and suppression of thioredoxin reductase 2 (Txnrd2), resulting in cell death [92,93]. The weak interaction between XBP1s and UPRE sequences in the promoter region of KLF9 likely suggests that KLF9 upregulation requires a stronger signal (high XBP1s levels) due to unmitigated stress, thereby serving as a forward feedback mechanism to induce cell death [92,93]. 

While the impact of spliced XBP1(s) has been extensively studied, much less is known about its unspliced counterpart, XBP1u. Our lack of knowledge regarding XBP1u is, in part, a consequence of the unstable nature of XBP1u (half-life of 11 min) [94]. Despite this, evidence suggesting a selective requirement for XBP1u in oxidative stress responses is available. Liu et al. demonstrated that MEFs lacking total XBP1 displayed elevated cell death in response to H_2_O_2_-induced oxidative stress compared to wild-type counterparts. Further analysis demonstrated the reduced induction of antioxidant genes including superoxide dismutase 1 (SOD1), thioredoxin 1 (TRX1) and catalase (Cat) in H_2_O_2_-treated XBP1 knockout MEFs. The selective reestablishment of XBP1u, but not XBP1s, restored the induction of antioxidant genes, underscoring a specific role for unspliced XBP1 in antioxidant responses [95]. A similar role for XBP1u has also been reported in endothelial cells, where selective overexpression of XBP1u increased survival following the induction of oxidative stress. Within this setting, XBP1u promotion of cell survival increased NRF2 stabilisation and HO-1 expression [96]. Collectively, these studies suggest that XBP1u contributes to responses by antioxidant genes important for decomposing O_2_^•−^ and H_2_O_2_. 

IRE1 signaling has also been linked to the control of ROS production via the upregulation of thioredoxin inhibiting protein (TXNIP). TXNIP acts as an inhibitor of the key antioxidant enzyme thioredoxin (TRX), with TXNIP inhibiting thioredoxin reductase activity by binding to its redox active domain [97]. A loss of TXNIP expression has been demonstrated to inhibit ROS production, whereas an overexpression of TXNIP has been shown to increase ROS [98]. Based on these observations, pathways controlling TXNIP expression may have central roles in regulating oxidative stress. Lerner and colleagues demonstrated that IRE1 signaling increases TXNIP levels during ER stress. Mechanistically, IRE1-mediated TXNIP regulation was linked to RIDD-facilitated degradation of miR-17, a micro RNA that normally suppresses TXNIP expression. The induction of ER stress in INS-1 cells rapidly increased TXNIP expression in an IRE1-dependent manner [99]. Elevated ROS, in addition to damaging proteins and lipids, can also promote inflammation by triggering the formation of the NLRP3 inflammasome [99]. Indeed, TXNIP has been demonstrated to interact with NLRP3 and, through this, promote inflammasome formation [100]. Therefore, IRE1 activity, in addition to controlling ROS levels via TXNIP regulation, may also have the ability to influence inflammasome formation [99]. 

### 4.3. ATF6 in Response to Oxidative Stress

Of the three UPR mediators, within the context of oxidative stress, the contribution of ATF6 is the least characterised. Nonetheless, Jin et al. identified a potential role for ATF6 in suppressing necrosis induced by increased ROS. The overexpression of ATF6 in neonatal rat ventricular myocytes (NRVM) treated with hydrogen peroxide, or induced mitochondrial ROS simulated via ischemia/reperfusion, increased cell viability relative to controls [101]. Further qPCR gene array analysis revealed 11 antioxidant genes regulated by ATF6, including catalase, peroxiredoxin 5 (PRDX5) and thioredoxin reductase 1 (TXNRD1), glutathione peroxidase 3 and 4 (GPx3, GPx4) and NAPDH quinone dehydrogenase 1 (NQO1). The selective activation of ATF6 with AA147 increased the expression of catalase and suppressed oxidative stress and apoptosis in mouse neurons following cardiac arrest. Interestingly, NRF2/HO-1 activation was also demonstrated as a feature of AA147 treatment [102]. Overall, these findings indicate an antioxidant role for ATF6.

## 5. Conclusions and Perspectives

**Figure 3:**
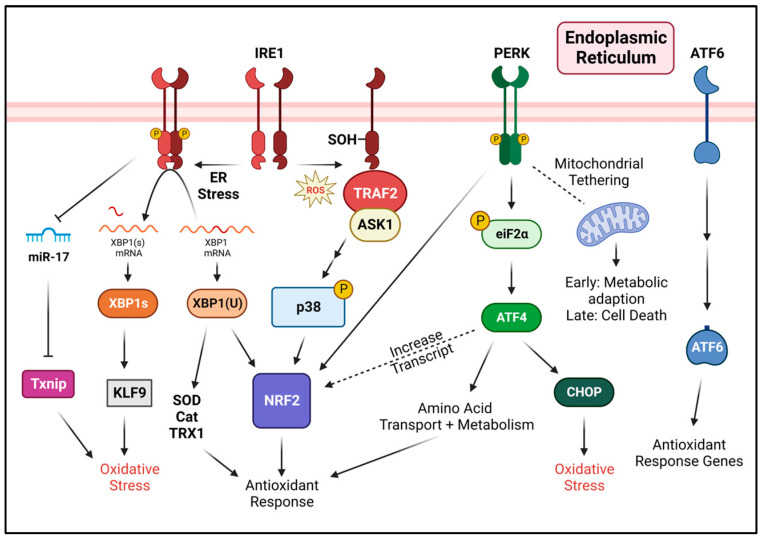
The three UPR signaling pathways (IRE1, PERK and ATF6) and their contribution to both antioxidant signaling and oxidative stress. Created with BioRender.com.

Classically, the UPR is considered a stress response system primarily aimed at resolving ER stress. However, the role of the ER in oxidative protein folding and its close association with mitochondria leaves the ER vulnerable to oxidative stress. Indeed, ER stress aggravates oxidative stress through increased protein folding and stimulated mitochondrial activity. As such, the UPR is also naturally equipped to deal with oxidative stress through the upregulation of antioxidant response genes to allow for initial adaptation (Figure 3). However, prolonged ER stress will initiate apoptosis in which some mechanisms are dependent on excessive ROS (oxidative stress) and mitochondrial crosstalk. Although the link between ER stress in ROS production has been established, the specific conditions for the ROS induction of ER stress, and, more specifically, UPR activation are not as well defined. The extent of UPR signaling appears to vary as some studies demonstrate activation by ROS [103], while others describe a limited, or lack of, activation [104]. Thus, further insights are required to elucidate the mechanisms by which ROS triggers ER stress and UPR activation. While the UPR is integral in maintaining ER homeostasis, it is amongst the many tools that the cell can utilize to adapt to stress. Indeed, other stress adaptation mechanisms, some of which are related to the UPR, can also aid in managing oxidative stress. Autophagy, a process that recycles damaged organelles including the ER and mitochondria, can supress oxidative stress and enable cell survival [105]. The maintenance of ER Ca^2+^ homeostasis and Ca^2+^ uptake through the sacro/endoplasmic reticulum calcium ATPase (SERCA) is also critical in preventing ER stress and elevated ROS levels [106]. Finally, heat shock proteins can be induced during oxidative stress to assist in protein folding and the degradation of damaged proteins [107,108]. Overall, understanding the interconnection amongst stress response pathways will provide a broader perspective on the progression of events that fine-tune the responses towards cellular adaptation and fate.
